# Real-World Tolvaptan Use in Autosomal Dominant Polycystic Kidney Disease: Insights from Two US Medical Centers

**DOI:** 10.34067/KID.0000000816

**Published:** 2025-04-22

**Authors:** Vinamratha Rao, Shahed Ammar, Abrar Alshorman, Michelle Fravel, Kerri A. McGreal, Franz T. Winklhofer, Lama Noureddine, Diana I. Jalal, Alan S.L. Yu, Reem A. Mustafa

**Affiliations:** 1 Department of Medicine, University of Kansas Medical Center, Kansas City, Kansas; 2 Department of Pharmacy Practice and Science, University of Iowa College of Pharmacy, Iowa City, Iowa; 3 Department of Pharmaceutical Care, University of Iowa Health Care, Iowa City, Iowa; 4 Department of Internal Medicine, University of Iowa Carver College of Medicine, Iowa City, Iowa; 5 Iowa City Veterans Affairs Health Care System, Iowa City, Iowa

**Keywords:** ADPKD, chronic kidney disease, clinical nephrology, cystic kidney, epidemiology and outcome, genetic renal disease, outcomes, polycystic kidney disease, vasopressin, cohort studies

## Abstract

**Key Points:**

- Twenty-seven percent of patients with autosomal dominant polycystic kidney disease discontinued tolvaptan in a real-world cohort in the midwestern United States.
- Most patients maintained tolvaptan on lower doses than trials, and a minority tolerated above the 45 mg (am)/15 mg (pm) starting dosage.
- Adverse effects, specifically aquaretic side effects, strongly influenced tolvaptan tolerability, dosage titration, and discontinuation.

**Background:**

Autosomal dominant polycystic kidney disease (ADPKD) is the most prevalent genetic kidney disease leading to kidney failure. Tolvaptan, a vasopressin V2 receptor antagonist, is the only medication approved by the US Food and Drug Administration for slowing kidney growth in individuals with rapidly progressive ADPKD, but its long-term tolerability and effective implementation has yet to be studied, particularly in real-world clinical settings within the United States.

**Methods:**

This retrospective cohort study examined adults with ADPKD treated with tolvaptan at the University of Kansas Medical Center and the University of Iowa Hospitals & Clinics from May 2018 to April 2023. Data on demographics, clinical characteristics, tolvaptan dosage, and treatment duration were collected from electronic health records for an average follow-up duration of 28.2 months (interquartile range: 8.5–47.1 months). The study focused on examining tolvaptan dosage trends, treatment discontinuation reasons, and the impact of aquaretic side effects on dosage and adherence.

**Results:**

Of 134 patients, 27% stopped tolvaptan during the observational period, with 10.4% of the cohort withdrawing from treatment due to intolerance of aquaretic side effects. Most patients maintained a lower tolvaptan dosage (≤45/15 mg) than in clinical trials, with two thirds of individuals who underwent dosage adjustment undergoing net decrease in dosage. Adverse effects significantly influenced and dosage decisions, presenting a potential early barrier for adherence, particularly in female patients.

**Conclusions:**

The study highlights real-world challenges in the use of tolvaptan for ADPKD, particularly for side effects leading to high discontinuation rates and dosage adjustments. These findings underscore the need for standardized and improved management strategies to enhance tolerability and adherence, offering insights for future research and practice in the treatment of ADPKD with tolvaptan.

## Visual Abstract

**Figure:**
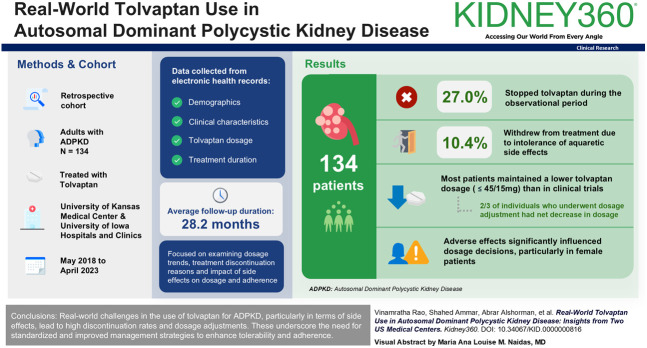

## Introduction

Autosomal dominant polycystic kidney disease (ADPKD) is the most common genetic kidney disease and a major cause of ESKD.^1^ ADPKD involves chronic growth of innumerable fluid-filled renal cysts, causing progressive loss of kidney function, massive kidney growth, hypertension, and several extrarenal complications.^2^ Multiplying systemic consequences from kidney expansion and ESKD undermine quality of life and necessitate considerable, life-long clinical management for individuals with ADPKD.^3,4^

Tolvaptan, an orally available selective vasopressin V2 receptor antagonist, is the only medication approved by the US Food and Drug Administration (FDA) to slow kidney function decline in rapidly progressive ADPKD.^3^ Two pivotal randomized clinical trials demonstrated its efficacy: the Tolvaptan Efficacy and Safety in Management of ADPKD and its Outcomes 3:4 (TEMPO 3:4) trial showed a 45% reduction in the rate of kidney growth and a 26% reduction in the slope of eGFR decline over 3 years in early-stage ADPKD,^5^ while the Replicating Evidence of Preserved Renal Function: An Investigation of Tolvaptan Safety and Efficacy in ADPKD (REPRISE) trial showed a 35% reduction in eGFR decline slope over 1 year in advanced ADPKD.^6^

Tolvaptan open-label extension studies,^7^ postmarketing surveillance,^8^ and treatment guidelines^9,10^ have demonstrated the drug's long-term benefits in slowing ADPKD progression. However, optimizing real-world tolvaptan use remains challenging due to factors including aquaretic side effects (polyuria, polydipsia, and nocturia), concerns for liver toxicity, and limited distribution through only three designated pharmacies in the United States.^5,6,9,11^ These challenges can significantly affect tolvaptan prescribing practices, tolerability, and adherence. Although a few studies such as the Canadian retrospective cohort at the Center for Innovative Management of PKD^12^ have begun to highlight these challenges, there is still a need for more comprehensive understanding of effective and sustainable tolvaptan implementation in diverse patient populations, particularly in the United States. In this study, we evaluate tolerability, dosing, and experience of tolvaptan use in a broad cohort of adults treated at two clinical centers in the midwestern United States. Our findings aim to motivate and inform nationwide multicenter studies to refine and optimize clinical management of ADPKD with tolvaptan.

## Methods

**Figure 1:**
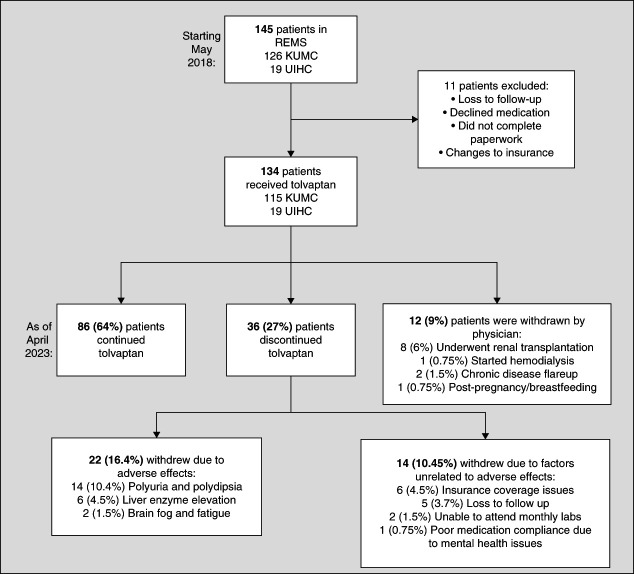
**Flow chart of patient selection, treatment outcomes, and treatment discontinuation reasons of the combined patient cohort (N=134).** “Chronic disease flareup” encompasses individuals who were withdrawn from treatment due to exacerbation of chronic illness other than ADPKD, such as ulcerative colitis. “Loss to follow-up” encompasses circumstances such as individuals moving state of residence, no longer pursuing treatment within the respective site's nephrology clinic, or no longer requesting refills of tolvaptan through their pharmacy. ADPKD, autosomal dominant polycystic kidney disease; KUMC, University of Kansas Medical Center; REMS, Risk Evaluation and Mitigation Strategy; UIHC, University of Iowa Hospitals & Clinics.

This retrospective study identified adults with ADPKD treated with tolvaptan in the outpatient nephrology clinics of the University of Kansas Medical Center (KUMC) and the University of Iowa Hospitals & Clinics (UIHC) from May 19, 2018, to April 1, 2023. After ethical clearance from the KUMC and UIHC Institutional Review Boards, we identified patients through the Risk Evaluation and Mitigation Strategy (REMS) program's lists of individuals prescribed tolvaptan by the study treating physicians. Patients enrolled in REMS completed paperwork documenting treatment indication and agreement to a liver function tests (LFT) to monitor potential liver toxicity. LFT monitoring mandated by the REMS program is conducted at baseline, 2 weeks after treatment initiation, 4 weeks later, monthly for the first 18 months of treatment, and then every 3 months thereafter. On REMS enrollment, tolvaptan (JYNARQUE) was prescribed and shipped to the patient by an approved specialty pharmacy after prior authorization. Medical insurance covered LFT testing to maintain REMS enrollment and continue treatment. To survey early treatment patterns, the study included patients documented in the electronic health record (EHR) to have taken tolvaptan for at least 1 day (Figure 1).

Data were collected using a structured prepiloted data abstraction form shared between institutions. V.R. and A.A. (KUMC) and A.S. (UIHC) abstracted each patient's EHR data on demographics and clinical information, encompassing age, sex, race and ethnicity, laboratory values, comorbidities, ADPKD family history, tolvaptan dosage, treatment duration, discontinuation reasons, and ADPKD Mayo/Irazabal Imaging Classification (MIC). Total kidney volume (TKV) was collected or computed from physician notes or historical imaging records, both using the kidney volume ellipsoid equation:^13^

kidney volume=length (average of sagittal and coronal lengths)×width×depth×(π/6)

Data were input to the Imaging Classification of ADPKD web-based calculator^13^ hosted by the Mayo Foundation and Medical Education and Research to calculate height-adjusted TKV and the MIC for stratifying ADPKD severity and risk of eGFR decline.^13–15^ Treating physicians reviewed data to ensure accuracy and reliability. REMS lists, data collection forms, and files containing patient identifiers were stored in secured clinical data servers. Only cleaned, deidentified datasets were used for descriptive analysis and visualization in Microsoft Excel and RStudio.

We analyzed demographics, clinical characteristics, and tolvaptan treatment characteristics (*e.g.*, dosage and discontinuation reason) using descriptive statistics for continuous variables (*e.g.*, age, laboratory values, and dosage) and frequencies for categorical variables (*e.g.*, sex, race and ethnicity, and adverse events). Chi-square testing (α=0.05) assessed differences in categorical variables, such as discontinuation rate among subgroups. Wilcoxon rank-sum tests compared treatment durations between withdrawn and ongoing patients by sex. A Kruskal-Wallis test assessed treatment duration across baseline age groups in the ongoing subgroup, followed by the Dunn test with Bonferroni correction for pairwise comparisons. Missing data were omitted before analysis, with the sample size (N) indicated in figure captions.

## Results

### Baseline Characteristics of Cohort

A total of 134 adults with ADPKD were treated with tolvaptan at KUMC (*n*=115) and UIHC (*n*=19) from May 2018 and April 2023. Of these, 126 individuals were registered in the KUMC REMS list and 19 individuals in the UIHC REMS list, with 11 individuals excluded from data abstraction due to loss to follow-up and declining tolvaptan (Figure 1). Baseline demographics and clinical characteristics were balanced between institutions (Supplemental Table 1), allowing a combined cohort. The combined cohort's baseline characteristics are presented in Table 1. The cohort was primarily White, with 10% encompassing Black, Asian, and Hispanic individuals. Most patients had hypertension (87%) and were treated with renin-angiotensin-aldosterone system inhibitors (80%). Among 40 biologically female individuals of reproductive age, seven used oral contraceptives or intrauterine devices. Patients had baseline CKD stage 1 (13%, eGFR≥90 ml/min), stage 2 (32%, eGFR=60–89 ml/min), stage 3a (28%, eGFR=45–59 ml/min), stage 3b (16%, eGFR=30–44 ml/min), and stage 4 (11%, eGFR=15–29 ml/min). Baseline imaging was available for 115 individuals to calculate an average height-adjusted TKV of 976.05±649.70 ml/m and classify 74% of the cohort into MIC stages 1C-1E (Table 1). A minority of patients in MIC 1A (*n*=1) and 1B (*n*=15) were treated with tolvaptan, including nine individuals with low eGFR for age (<65 ml/min for individuals 55 years or younger) and six individuals with large kidney length for age (>16.5 cm in patients younger than 50 years).^9,10^

**Table 1:** Baseline demographics and clinical characteristics of the combined patient cohort from the University of Kansas Medical Center and University of Iowa Hospital and Clinics (N=134)

Characteristics	Statistic (N=134)
Average age at starting tolvaptan (yr)	44.4±12.3
Female	70 (52%)
Average height (cm)	175.1±10.6
Average body mass index (kg/m^2^)	29.7±6.6
Black or African American/non-Hispanic, Latine, or Spanish origin	6 (4%)
Other/Hispanic, Latine, or Spanish origin	5 (4%)
Other/non-Hispanic, Latine or Spanish origin, or declined	3 (2%)
White or Caucasian/non-Hispanic, Latine, or Spanish origin	120 (90%)
**Family history of ADPKD**	
Yes	108 (81%)
No	17 (13%)
Unsure	9 (7%)
**Comorbidities**	
Hypertension	116 (87%)
Liver cysts	58 (43%)
Brain aneurysms	5 (4%)
Diabetes mellitus	4 (3%)
Heart disease	15 (11%)
**Concomitant medications**	
RAASI	107 (80%)
Beta blockers	42 (31%)
Statins	34 (25%)
Diuretics	20 (15%)
Calcium channel blockers/other antihypertensives	19 (14%)
Diabetes medications	8 (6%)
SGLT inhibitors	2 (1%)
**CKD stage**	
1	18 (13%)
2	43 (32%)
3a	37 (28%)
3b	21 (16%)
4	15 (11%)
**Mayo ADPKD imaging classification**	
1A	1 (1%)
1B	15 (11%)
1C	52 (39%)
1D	27 (20%)
1E	20 (15%)
Baseline imaging not available	19 (14%)
**Urologic symptoms of PKD at baseline**	
Kidney pain episodes	25 (19%)
Cyst rupture/infection	9 (7%)
UTI	10 (7%)

ADPKD, autosomal dominant polycystic kidney disease; PKD, Polycystic Kidney Disease; RAASI, renin-angiotensin-aldosterone system inhibitors; SGLT, sodium-glucose transporter; UTI, urinary tract infection.

### Tolvaptan Discontinuation

By the end of the study, 86 of the 134 patients in the cohort (64%) were still undergoing tolvaptan treatment, whereas 12 patients (9%) were withdrawn from tolvaptan by their physician and 36 patients (27%) personally withdrew from tolvaptan treatment over a median follow-up of 28.2 months (interquartile range [IQR]: 8.5–47.1 months). Figure 1 shows two discontinuation categories for the 36 individuals who withdrew: (*1*) adverse effects attributed to tolvaptan treatment and (*2*) factors unrelated to tolvaptan adverse effects. The three most common reasons for discontinuation were difficulty tolerating aquaretic side effects (14 patients, 10.4%), increase in LFTs (6 patients, 4.5%), and loss of health insurance coverage (6 patients, 4.5%). Of the six patients who discontinued tolvaptan because of significantly elevated LFTs, four exhibited notable aminotransferase elevations below two times the upper limit of normal (ULN). Two patients experienced aminotransferase elevations ≥3× ULN, fulfilling JYNARQUE FDA drug label criteria for permanent discontinuation.^16–18^ Notably, none of the six patients had concurrent liver conditions or a history of liver disease, and all experienced LFT normalization after discontinuation.

**Figure 2:**
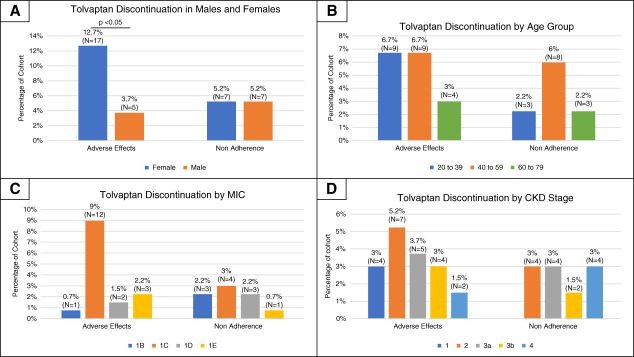
**Tolvaptan discontinuation rates by subgroup.** Rates are displayed within (A) sex, (B) age group, (C) MIC, and (D) CKD Stage for the combined cohort (*N*=134). In (A), chi-square testing showed a significantly higher adverse effect discontinuation rate for female patients compared with male patients (*P* = 0.01437). MIC, ADPKD Mayo/Irazabal Imaging Classification.

Figure 2 shows discontinuation rates by sex, age, MIC, and CKD stage. Female patients had higher discontinuation due to adverse effects (12.7%, *n*=17), compared with male patients (3.7%, *n*=5). Although discontinuation due to polyuria and polydipsia was higher among female patients (8%, *n*=11) than male patients (2%, *n*=3), this difference was not statistically significant (*P* = 0.055). The small sample size limits definitive conclusions on sex difference in tolerability without further study.

### Tolvaptan Dosing Trends

Most patients (89%) started tolvaptan with 45 mg in the morning and 15 mg 8 hours later. Only 9% (12 of 134 patients) started with higher doses (60/30 or 90/30 mg), including eight individuals who participated in tolvaptan clinical trials. As seen in Table 2, the 45/15 mg dosage accounted for most ongoing doses (76%) and ending doses before discontinuation by patient or physician (60%). The majority (68%, 91) maintained the same dose, specifically the starting 45/15 mg dosage (62%, 83). Among those requiring dosage change (32%, 43), the majority had a net decrease in dosage (67%, 29 of 43 patients).

**Table 2:** Characteristics of tolvaptan treatment and adverse events of combined cohort (N=134)

Characteristics	Statistic (N=134)
**Dosing trend**	
Maintained on same dose	91 (68%)
Required multiple changes to dose	43 (32%)
**Starting tolvaptan dose**	
45 mg (am)/15 mg (pm)	119^a^ (89%)
>45 mg (am)/15 mg (pm)	12^a^ (9%)
<45 mg (am)/15 mg (pm)	3 (2%)
**Ongoing tolvaptan dose**	
45 mg (am)/15 mg (pm)	65^a^ (48%)
>45 mg (am)/15 mg (pm)	8^a^ (6%)
<45 mg (am)/15 mg (pm)	13 (10%)
**Stopping tolvaptan dose**	
45 mg (am)/15 mg (pm)	28^a^ (21%)
>45 mg (am)/15 mg (pm)	12^a^ (9%)
<45 mg (am)/15 mg (pm)	8 (6%)
Average fluid intake during tolvaptan (L) (*n*=86)	4.89±2.14
Average frequency of nocturia during tolvaptan (*n*=75)	2.21±0.96
**Urologic symptoms during treatment**	
Kidney pain episodes	32 (24%)
Cyst rupture and/or infection	15 (11%)
Urinary tract infection	15 (11%)
**Discontinuation due to liver enzyme elevations**	
ALT or AST elevation <2× ULN	4 (3%)
ALT or AST elevation ≥3× ULN	2 (1.5%)

ALT, alanine aminotransferase; AST, aspartate aminotransferase; ULN, upper limit of normal.

a Signifies inclusion of prior tolvaptan clinical trial participants (*n*=8 [starting dose: 45/15 mg=2 and >45/15 mg=6; ongoing dose: 45/15 mg=4 and >45/15 mg=2; stopping dose: 45/15 mg=1 and >45/15 mg=1]).

Among the 12 patients who discontinued higher doses (60/30 mg and 90/30 mg), the three major reasons for discontinuation were aquaretic side effects (4, 3%), renal transplantation (3, 2.2%), and LFT elevation <2× ULN (2, 1.5%). Eight patients (6%) experienced intermittent tolvaptan treatment due to reasons such as insurance loss, severe aquaretic side effects, difficulty complying with monthly laboratory results due to travel, and gastrointestinal illness. Of the 20 patients who concurrently took diuretics and sodium-glucose cotransporter-2 inhibitors, 15 continued tolvaptan, while 5 withdrew due to factors unrelated to tolvaptan effects.

### Treatment Duration Analysis

**Figure 3:**
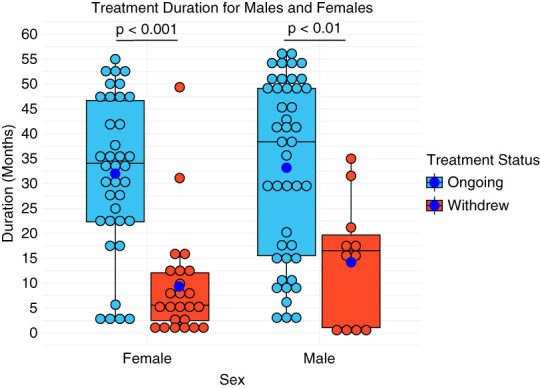
**Time in months spent on tolvaptan based on sex (*n*=116), excluding patients withdrawn from treatment by physicians for reasons not attributable to tolvaptan (*n*=12) and tolvaptan clinical trial participants (*n*=8).** Blue points represent average duration per group. Treatment Status: “Ongoing” represents patients who have continued treatment beyond the study period end (April 2023), while “Withdrew” represents patients who stopped treatment due to adverse effects or nonadherence factors within May 2018–April 2023. Wilcoxon rank-sum tests show significantly shorter treatment durations for those who withdrew in both women (*P* = 1.661e-06) and men (*P* = 0.0036).

The median treatment duration was 4.85 months (IQR: 1.91–12.4 months; *n*=22) among those who discontinued due to adverse effects (polyuria/polydipsia, increased LFTs, brain fog/fatigue), 12.8 months (IQR: 5.9–16.5 months; *n*=13) for those who discontinued due to factors unrelated to tolvaptan's effects, and 34.6 months (IQR: 18.2–48.7 months; *n*=81) for patients who continued treatment. Eight clinical trial participants who had received tolvaptan before observation were excluded only from treatment duration analysis. Figure 3 shows the duration of tolvaptan treatment in months for patients who continued or withdrew treatment, divided by sex. Naturally, treatment duration was significantly shorter for those who withdrew compared with those who continued, with more pronounced difference in female patients (*P* < 0.001, *n*=61) than male patients (*P* < 0.01; *n*=55). However, there was no significant difference in treatment duration for those who withdrew.

## Discussion

Our study evaluated tolvaptan's real-world safety, adherence, and outcomes in a retrospective cohort of adults with ADPKD treated with tolvaptan at two major Midwestern US medical centers. Over a median follow-up of 28.2 months, 27% of patients withdrew treatment. Most started and maintained a 45/15 mg dosage; among those who adjusted doses, two thirds had a net decrease. The most common stand-alone discontinuation reason was intolerance of aquaretic side effects, while treatment interruptions were driven by external factors such as insurance changes. Female patients had a higher discontinuation rate due to adverse effects and more significant treatment duration difference between those who discontinued treatment compared with who continued treatment.

### Real-World Tolvaptan Discontinuation

The proportion of patients who withdrew from tolvaptan (36 out of 134, 27%) in our real-world study mirrors the withdrawal rates seen in TEMPO 3:4 (23%) and REPRISE (15%) cohorts.^5,6^ The withdrawal rate due to aquaretic side effects in our cohort was 10.4%, falling between TEMPO (15.4%) and REPRISE (8.37%). However, most patients in our cohort started with the recommended 45/15 mg dose,^9,10,19,20^ but only 15% (20 of 134) escalated to higher doses (60/30 or 90/30 mg), compared with 76% in TEMPO and 90.5% in REPRISE.^5,6^ Both clinical trials enriched participant selection for individuals who could tolerate tolvaptan's aquaretic side effects. In the TEMPO 3:4 trial, participants had to affirm their ability to tolerate the assigned tolvaptan dose, while REPRISE participants underwent an 8-week tolvaptan run-in period, which included dose adjustment phases and a tolerance assessment of the maximum daily doses (60/30 or 90/30 mg) to ensure side effect tolerability before randomization and the double-blind phase.^5,6^

Such rigorous selection processes are important to note when comparing clinical trial outcomes to our real-world cohort, in which patients faced fewer prescreening measures for tolerability. Informed consent in our cohort included discussions about aquaretic side effects, and similar to the Canadian observational study at the Center for Innovative Management of Polycystic Kidney Disease (PKD),^12^ some MIC 1C-1E patients declined to start tolvaptan due to aquaretic concerns. This suggests positive selection bias toward patients willing to tolerate tolvaptan's side effects, although with less formal selection in real-world settings.

Treatment discontinuation due to side effects narrowly exceeded discontinuation due to factors unrelated to the medication's effects. Potential barriers to tolvaptan adherence, such as loss of insurance coverage, contributed to intermittent treatment (*n*=4) or withdrawal (*n*=6). Limited, variable notation regarding patient financial circumstances limits the analysis scope in this article. However, available records indicate that patients who lost insurance coverage due to employment changes or aging into Medicare (65 years or older) could not afford out-of-pocket costs for tolvaptan and frequent LFT testing required by the REMS program. The average cost for 10 oral tablets of 15 mg tolvaptan is $4482 USD,^21^ which could significantly influence tolvaptan prescription for individuals lacking stable insurance coverage and older patients seeking to continue tolvaptan beyond age 65 years. Future evaluation into the financial barriers that influence initial prescribing decisions and the factors that facilitate or prevent insurance coverage of tolvaptan is warranted.

### Dosage Titration and Tolerability

Dosage adjustments were driven by patient-reported intolerance of aquaretic side effects, contributing to frequent down-titration, sometimes to doses below clinical trial doses—as low as 22.5/7.5 mg or even 15 mg daily (Supplemental Table 2). Although unknown to be therapeutic, low doses were used to continue therapy for patients unable to tolerate higher doses and offer some potential benefit over discontinuation.

Study physicians documented efforts to improve tolerability by counseling patients on strategies such as a low-solute diet and limiting water intake before sleep, aiming to gradually increase dosage as tolerated. This reflects the challenge of balancing tolerability with maintaining tolvaptan efficacy, described as keeping urine osmolality below 280–300 mOsm/kg to maintain hypotonic urine compared with plasma.^5,6^ Although physician adherence to urine osmolality testing varied initially, most study physicians now aim to maintain patient osmolality <280 mOsm/kg. Ideally, urine osmolality should be measured before the morning dose. Although no formal urine osmolality testing protocol was in place during the study period, available records show predrug urine samples were tested in some patients, highlighting variability in practice that needs to be measured to inform treatment standardization and guideline development. Until recently, longitudinal tolvaptan implementation studies have largely overlooked urine osmolality testing, dosing variability, sustainable dosing, and side effect mitigation counseling in routine clinical practice.^22^

### Liver Enzyme Elevation during Tolvaptan Treatment

Clinicians discontinued treatment for all six patients (4.5%) who experienced significant LFT elevations during tolvaptan therapy. Of these, only two patients (1.5%) had LFT elevations ≥3 times the ULN, meeting tolvaptan's FDA drug label criteria for immediate discontinuation.^16–18^ The remaining four patients had LFT elevations <2× ULN, which led to discontinuation and subsequent LFT normalization. These findings align with the TEMPO 3:4 and REPRISE trials, where 4.4% and 5.6% of participants, respectively, had alanine aminotransferase increases >3× ULN, leading to treatment interruption or discontinuation. LFT elevations generally occurred within the first 18 months of treatment and resolved after discontinuation. Similarly, postmarketing surveillance of 6711 patients found fewer than 1% experienced possible severe drug-induced liver injury, highlighting the value of REMS monitoring for early detection of liver safety concerns.^23^ Despite the FDA drug label allowing reinitiation with increased monitoring after LFT normalization, clinicians in our cohort chose not to reinitiate therapy. Although this decision introduces potential bias—since some patients may have tolerated continued therapy with monitoring—it also reflects clinical caution and variability in real-world practice.

### Baseline Kidney Volumetry for Risk Assessment

The FDA's approval of tolvaptan for rapidly progressive ADPKD, as defined by international consensus statements, includes patients with MIC Classes 1C-1E who are at higher risk of early progression to renal failure.^9,10,13,19^ In our observational cohort, most patients fell within MIC 1C-1E, while a minority in Classes 1A-1B were treated with tolvaptan due to low eGFR and/or large kidney length for age. Nineteen patients lacked usable imaging to estimate TKV and MIC, and many available magnetic resonance images predated tolvaptan eligibility consideration by ≥2 years. Kidney length measurements from historical ultrasound imaging and eGFR trends aided risk assessment when acceptable imaging to calculate MIC was unavailable. Clinicians manually measured kidney dimensions in axial, sagittal, and coronal views to estimate TKV using the ellipsoid equation,^13^ rather than more precise but expensive and time-intensive methods such as planimetry and stereology.^24–28^ Accurate baseline TKV and MIC computed from relatively recent imaging, when available, were critical for determining treatment eligibility and securing insurance approval for tolvaptan decisions that chiefly depended on the patient's estimated risk of rapid ADPKD progression.

### Treatment Duration and Discontinuation in Different Groups

Adverse effects such as frequent urination and thirst tend to disproportionately affect female patients,^5,6^ contributing to higher discontinuation—warranting further investigation in a larger real-world cohort. In our study, female patients demonstrated a significantly higher discontinuation due to adverse effects than male patients, although differences specific to polyuria and polydipsia were less pronounced. Female patients who withdrew had significantly shorter treatment durations, suggesting potential sex-based differences in tolerance. Subgroup analysis by age, CKD stage, and MIC showed no significant differences in discontinuation rates or treatment duration; however, interpretation was limited by small subgroup sizes (*n*<5). As this is an exploratory analysis assessing potential subgroup effects, findings should be interpreted with caution due to limited statistical power. Future multicenter studies are needed to enable more robust subgroup analyses.

### Addressing Real-World Implementation Challenges

Our study highlights the challenges of translating clinical trial findings into real-world practice. Nonstandard decision making, such as lower dosing regimens and lack of tolvaptan rechallenge after LFT normalization, was observed and likely reflects both patient-reported discomfort with aquaretic side effects and provider caution. Although efforts to counsel patients on side effect mitigation and dosage adjustments were documented in clinician notes, the observed varying practice across physicians and institutions underscores the need to address both patient tolerance and clinician confidence toward tolvaptan use.

Standardized guidelines and training surrounding symptom management strategies and tailored dosing protocols can both enhance patients' tolerability of tolvaptan and support clinicians in managing tolvaptan therapy effectively. Proactive patient education on managing aquaretic side effects, gradual individualized dose titration, multidisciplinary support involving dietitians and pharmacists, and personalized follow-up regarding medication tolerance could significantly improve real-world patient outcomes and treatment adherence.

### Study Strengths and Limitations

A notable strength of this study is its real-world design, incorporating a diverse range of disease severities and health care experiences in two major US medical centers, capturing a snapshot of tolvaptan's tolerability and implementation in the Midwestern pocket of the ADPKD population, laying the groundwork for a nation-wide survey tolvaptan use and effectiveness. Despite inherent selection bias in observational cohorts, we included all patients who took tolvaptan for at least 1 day to capture early treatment adherence barriers. The longitudinal 5-year follow-up allowed observation of long-term outcomes and treatment adherence, which is critical for chronically progressive diseases such as ADPKD. By focusing on specific dosing regimens and patient adherence, this study offers valuable insights for future research on practical ADPKD management.

Some limitations of this study include its observational design and limited sample size, which hinder establishing firm causal links between patient characteristics, treatment patterns, and outcomes. Challenges with incomplete EHRs and follow-up variability affect data accuracy and reliability. In addition, the study is limited to geographically distinct participants and a small number of treating physicians within two Midwestern US medical centers, which limits the generalizability of findings to all adults with ADPKD and global clinical practice patterns.

### Future Studies and Conclusion

To strengthen the applicability of these findings, we are expanding the cohort through collaboration with additional medical centers and PKD Centers of Excellence across the United States. This broader geographical inclusion will enable a socioeconomically and ethnically diverse cohort that is (*1*) generalizable to the adult ADPKD population and (*2*) statistically powered for robust subgroup analyses of tolvaptan use and effectiveness across sex, age, and disease severity in the United States. A key objective is to assess effectiveness of lower clinical tolvaptan dosages compared with clinical trial doses. We will compare longitudinal outcomes in tolvaptan-treated patients with matched individuals who have not received tolvaptan, considering demographics, ADPKD severity, and treatment site. Key outcomes will include changes in eGFR, TKV, urine osmolality, incidence of pain and urologic complications of PKD, and time to initiation of KRT.

Given the variability in prescribed dosages and low maintenance doses observed, we plan to use mixed method surveys to (*1*) assess clinician confidence in prescribing and maintaining tolvaptan treatment and (*2*) gather clinician strategies used by clinicians to mitigate side effects and improve treatment adherence. In addition, we will investigate outcomes of treated MIC 1A-1B patients to explore potential benefits of tolvaptan in this group deemed lower risk. Building on multicenter data and existing guidelines, we aim to develop an updated protocol for tolvaptan prescription, side effect mitigation, and efficacy monitoring. These efforts can optimize treatment adherence and improve clinical outcomes across diverse patient populations.

In summary, this study enhances our understanding of ADPKD management with tolvaptan in clinical practice, identifies key areas for improving adherence, and lays the groundwork for future research in an expanded nationwide cohort. Addressing these gaps is essential for optimizing treatment strategies and improving outcomes for individuals with rapidly progressive ADPKD.

## Supplementary Material

## Data Availability

All data are included in the manuscript and/or supporting information; Partial restrictions to the data and/or materials apply; This data will be available upon request by email to the corresponding author, Dr. Reem Mustafa (rmustafa@kumc.edu).

## Supplemental Material

Supplemental Table 1: Baseline demographics and clinical characteristics of the separate University of Kansas Medical Center (KUMC) and University of Iowa Hospital and Clinics (UIHC) cohorts.

Supplemental Table 2: Tolvaptan dosing trends and treatment withdrawal reasons of the separate cohorts.

Supplemental Table 3: Stopping dose and reason for withdrawing from tolvaptan treatment.
