# Supplementary material for: Real-World Tolvaptan Use in Autosomal Dominant Polycystic Kidney Disease: Insights from Two US Medical Centers
Source: Kidney360. 2025 Apr 22;6(9):1522–31. doi: 10.34067/KID.0000000816 (PMC12483031; doi:10.34067/KID.0000000816)
Supplement: Supplementary file 1 [file kidney360-6-01522-s001.pdf]

ASN American Society of Nephrology
JASN Journal of the American Society of Nephrology
KIDNEY360 Accessing Our World From Every Angle
CJASN Clinical Journal of the American Society of Nephrology

ASN Journal Disclosure Form

As per ASN journal policy, I have disclosed any financial relationships or commitments I have held in the past 36 months as included below. I have listed my Current Employer below to indicate there is a relationship requiring disclosure. If no relationship exists, my Current Employer is not listed.

A. Alshorman has nothing to disclose.

I understand that the information above will be published within the journal article, if accepted, and that failure to comply and/or to accurately and completely report the potential financial conflicts of interest could lead to the following: 1) Prior to publication, article rejection, or 2) Post-publication, sanctions ranging from, but not limited to, issuing a correction, reporting the inaccurate information to the authors' institution, banning authors from submitting work to ASN journals for varying lengths of time, and/or retraction of the published work.

Name: Abrar Alshorman
Manuscript ID: K360-2024-000519R2
Manuscript Title: Real-World Tolvaptan Use in Autosomal Dominant Polycystic Kidney Disease: Insights from Two U.S Medical Center
Date of Completion: February 5, 2025
Disclosure Updated Date: October 19, 2024

ASN American Society of Nephrology
JASN Journal of the American Society of Nephrology
KIDNEY360 Accessing Our World From Every Angle
CJASN Clinical Journal of the American Society of Nephrology

ASN Journal Disclosure Form

As per ASN journal policy, I have disclosed any financial relationships or commitments I have held in the past 36 months as included below. I have listed my Current Employer below to indicate there is a relationship requiring disclosure. If no relationship exists, my Current Employer is not listed.

S. Ammar reports the following:
Employer: University of Iowa Hospitals & Clinics

I understand that the information above will be published within the journal article, if accepted, and that failure to comply and/or to accurately and completely report the potential financial conflicts of interest could lead to the following: 1) Prior to publication, article rejection, or 2) Post-publication, sanctions ranging from, but not limited to, issuing a correction, reporting the inaccurate information to the authors' institution, banning authors from submitting work to ASN journals for varying lengths of time, and/or retraction of the published work.

Name: Shahed Ammar
Manuscript ID: K360-2024-000519R1
Manuscript Title: Real-World Tolvaptan Use in Autosomal Dominant Polycystic Kidney Disease: Insights from Two U.S Medical Centers
Date of Completion: October 21, 2024
Disclosure Updated Date: October 21, 2024

ASN American Society of Nephrology | JASN Journal of the American Society of Nephrology | KIDNEY360 Accessing Our World From Every Angle | CJASN Clinical Journal of the American Society of Nephrology

ASN Journal Disclosure Form

As per ASN journal policy, I have disclosed any financial relationships or commitments I have held in the past 36 months as included below. I have listed my Current Employer below to indicate there is a relationship requiring disclosure. If no relationship exists, my Current Employer is not listed.

M. Fravel reports the following:
Consultancy: Lex-Comp

I understand that the information above will be published within the journal article, if accepted, and that failure to comply and/or to accurately and completely report the potential financial conflicts of interest could lead to the following: 1) Prior to publication, article rejection, or 2) Post-publication, sanctions ranging from, but not limited to, issuing a correction, reporting the inaccurate information to the authors' institution, banning authors from submitting work to ASN journals for varying lengths of time, and/or retraction of the published work.

Name: Michelle A. Fravel
Manuscript ID: https://www.asn-online.org/myasn/profile.aspx
Manuscript Title: Real-World Tolvaptan Use in Autosomal Dominant Polycystic Kidney Disease: Insights from Two U.S Medical Centers
Date of Completion: February 4, 2025
Disclosure Updated Date: February 4, 2025

ASN American Society of Nephrology JASN Journal of the American Society of Nephrology KIDNEY360 Accessing Our World From Every Angle CJASN Clinical Journal of the American Society of Nephrology

ASN Journal Disclosure Form

As per ASN journal policy, I have disclosed any financial relationships or commitments I have held in the past 36 months as included below. I have listed my Current Employer below to indicate there is a relationship requiring disclosure. If no relationship exists, my Current Employer is not listed.

D. Jalal reports the following:
Employer: University of Iowa; Iowa City VA; Consultancy: Meridian Health Comms; Research Funding: AstraZenica; Corvidia;; Honoraria: K-INBRE; Reata; Sullivan conference- University of Kansas; PER (Physicians Education Resource); and Advisory or Leadership Role: Reata; CSL Behring.

I understand that the information above will be published within the journal article, if accepted, and that failure to comply and/or to accurately and completely report the potential financial conflicts of interest could lead to the following: 1) Prior to publication, article rejection, or 2) Post-publication, sanctions ranging from, but not limited to, issuing a correction, reporting the inaccurate information to the authors' institution, banning authors from submitting work to ASN journals for varying lengths of time, and/or retraction of the published work.

Name: Diana I. Jalal
Manuscript ID: K360-2024-000519R1
Manuscript Title: Real-World Tolvaptan Use in Autosomal Dominant Polycystic Kidney Disease: Insights from Two U.S Medical Centers
Date of Completion: October 26, 2024
Disclosure Updated Date: July 15, 2024

ASN American Society of Nephrology JASN Journal of the American Society of Nephrology KIDNEY360 Accessing Our World From Every Angle CJASN Clinical Journal of the American Society of Nephrology

ASN Journal Disclosure Form

As per ASN journal policy, I have disclosed any financial relationships or commitments I have held in the past 36 months as included below. I have listed my Current Employer below to indicate there is a relationship requiring disclosure. If no relationship exists, my Current Employer is not listed.

K. McGreal reports the following:
Employer: University of Kansas School of Medicine

I understand that the information above will be published within the journal article, if accepted, and that failure to comply and/or to accurately and completely report the potential financial conflicts of interest could lead to the following: 1) Prior to publication, article rejection, or 2) Post-publication, sanctions ranging from, but not limited to, issuing a correction, reporting the inaccurate information to the authors' institution, banning authors from submitting work to ASN journals for varying lengths of time, and/or retraction of the published work.

Name: Kerri A. McGreal
Manuscript ID: K360-2024-00062+R1
Manuscript Title: Real-World Tolvaptan Use in Autosomal Dominant Polycystic Kidney Disease: Insights From Two U.S. Medical Centers
Date of Completion: October 23, 2024
Disclosure Updated Date: October 23, 2024

ASN American Society of Nephrology
JASN Journal of the American Society of Nephrology
KIDNEY360 Accessing Our World From Every Angle
CJASN Clinical Journal of the American Society of Nephrology

ASN Journal Disclosure Form

As per ASN journal policy, I have disclosed any financial relationships or commitments I have held in the past 36 months as included below. I have listed my Current Employer below to indicate there is a relationship requiring disclosure. If no relationship exists, my Current Employer is not listed.

R. Mustafa reports the following:
Employer: University of Kansas; Ownership Interest: Yes, for multiple stocks that are not health related. The following are health related (BrainCheck, HealthJoy, IVX); Advisory or Leadership Role: Member-Board of the director for Evidence Foundation; member-The Midwest Comparative Effectiveness Public Advisory Council (Midwest CEPAC) convened by The Institute for Economic and Economic Reviews (ICER).; Advisory board for the renal round table, NKF Midwest; Counselor-Women In Nephrology (WIN).; and Other Interests or Relationships: Chair-the KDIGO methods committee and member of the KDIGO executive committee; Member-The Canadian Society of Nephrology Clinical Practice Guidelines Committee ; Member-The GRADE guidance group.

I understand that the information above will be published within the journal article, if accepted, and that failure to comply and/or to accurately and completely report the potential financial conflicts of interest could lead to the following: 1) Prior to publication, article rejection, or 2) Post-publication, sanctions ranging from, but not limited to, issuing a correction, reporting the inaccurate information to the authors' institution, banning authors from submitting work to ASN journals for varying lengths of time, and/or retraction of the published work.

Name: Reem Mustafa
Manuscript ID: K360-2024-000519R2
Manuscript Title: Real-World Tolvaptan Use in Autosomal Dominant Polycystic Kidney Disease: Insights from Two U.S Medical Centers
Date of Completion: March 27, 2025
Disclosure Updated Date: March 27, 2025

ASN Journal Disclosure Form

As per ASN journal policy, I have disclosed any financial relationships or commitments I have held in the past 36 months as included below. I have listed my Current Employer below to indicate there is a relationship requiring disclosure. If no relationship exists, my Current Employer is not listed.

L. Noureddine reports the following:
Employer: Universty of Iowa Carver College of Medicine

I understand that the information above will be published within the journal article, if accepted, and that failure to comply and/or to accurately and completely report the potential financial conflicts of interest could lead to the following: 1) Prior to publication, article rejection, or 2) Post-publication, sanctions ranging from, but not limited to, issuing a correction, reporting the inaccurate information to the authors' institution, banning authors from submitting work to ASN journals for varying lengths of time, and/or retraction of the published work.

Name: Lama A. Noureddine
Manuscript ID: K360-2024-000519R2
Manuscript Title: "Real-World Tolvaptan Use in Autosomal Dominant Polycystic Kidney Disease: Insights from Two U.S Medical Centers,"
Date of Completion: April 4, 2025
Disclosure Updated Date: April 4, 2025

ASN
American Society of Nephrology
JASN
Journal of the American Society of Nephrology
KIDNEY360
Accessing Our World From Every Angle
CJASN
Clinical Journal of the American Society of Nephrology

ASN Journal Disclosure Form

As per ASN journal policy, I have disclosed any financial relationships or commitments I have held in the past 36 months as included below. I have listed my Current Employer below to indicate there is a relationship requiring disclosure. If no relationship exists, my Current Employer is not listed.

V. Rao has nothing to disclose.

I understand that the information above will be published within the journal article, if accepted, and that failure to comply and/or to accurately and completely report the potential financial conflicts of interest could lead to the following: 1) Prior to publication, article rejection, or 2) Post-publication, sanctions ranging from, but not limited to, issuing a correction, reporting the inaccurate information to the authors' institution, banning authors from submitting work to ASN journals for varying lengths of time, and/or retraction of the published work.

Name: Vinamratha Rao
Manuscript ID: K360-2024-000519R1
Manuscript Title: Real-World Tolvaptan Use in Autosomal Dominant Polycystic Kidney Disease: Insights from Two U.S Medical Centers
Date of Completion: October 21, 2024
Disclosure Updated Date: May 13, 2024

ASN
American Society of Nephrology
JASN
Journal of the American Society of Nephrology
KIDNEY360
Accessing Our World From Every Angle
CJASN
Clinical Journal of the American Society of Nephrology

ASN Journal Disclosure Form

As per ASN journal policy, I have disclosed any financial relationships or commitments I have held in the past 36 months as included below. I have listed my Current Employer below to indicate there is a relationship requiring disclosure. If no relationship exists, my Current Employer is not listed.

F. Winklhofer reports the following:
Employer: KUMC

I understand that the information above will be published within the journal article, if accepted, and that failure to comply and/or to accurately and completely report the potential financial conflicts of interest could lead to the following: 1) Prior to publication, article rejection, or 2) Post-publication, sanctions ranging from, but not limited to, issuing a correction, reporting the inaccurate information to the authors' institution, banning authors from submitting work to ASN journals for varying lengths of time, and/or retraction of the published work.

Name: Franz Winklhofer
Manuscript ID: K360-2024-000519R1
Manuscript Title: Real-World Tolvaptan Use in Autosomal Dominant Polycystic Kidney Disease: Insights from Two U.S Medical Centers
Date of Completion: December 2, 2024
Disclosure Updated Date: December 2, 2024

ASN American Society of Nephrology | JASN Journal of the American Society of Nephrology | KIDNEY360 Accessing Our World From Every Angle | CJASN Clinical Journal of the American Society of Nephrology

ASN Journal Disclosure Form

As per ASN journal policy, I have disclosed any financial relationships or commitments I have held in the past 36 months as included below. I have listed my Current Employer below to indicate there is a relationship requiring disclosure. If no relationship exists, my Current Employer is not listed.

A. Yu reports the following:
Employer: University of Kansas Medical Center; University of Kansas Health System; Consultancy: Regulus, Calico; Ownership Interest: Pfizer, Dialysis Associates; Research Funding: Regulus; Honoraria: Elsevier, Wolters Kluwer; Advisory or Leadership Role: Paid advisory board: Regulus, Calico, Sarepta, Johnson & Johnson, Travere; and Other Interests or Relationships: PKD Foundation Scientific Advisory Board; JASN Deputy Editor.

I understand that the information above will be published within the journal article, if accepted, and that failure to comply and/or to accurately and completely report the potential financial conflicts of interest could lead to the following: 1) Prior to publication, article rejection, or 2) Post-publication, sanctions ranging from, but not limited to, issuing a correction, reporting the inaccurate information to the authors' institution, banning authors from submitting work to ASN journals for varying lengths of time, and/or retraction of the published work.

Name: Alan S.L. Yu
Manuscript ID: K360-2024-000519R2
Manuscript Title: Real-World Tolvaptan Use in Autosomal Dominant Polycystic Kidney Disease: Insights from Two U.S Medical Centers
Date of Completion: February 5, 2025
Disclosure Updated Date: February 5, 2025
