# Supplementary material for: Real-World Tolvaptan Use in Autosomal Dominant Polycystic Kidney Disease: Insights from Two US Medical Centers
Source: Kidney360. 2025 Apr 22;6(9):1522–31. doi: 10.34067/KID.0000000816 (PMC12483031; doi:10.34067/KID.0000000816)
Supplement: Supplementary file 2 [file kidney360-6-01522-s002.pdf]

Supplemental Material to: “Real-World Tolvaptan Use for ADPKD in the United States”

1) **Supplemental Table 1**
2) **Supplemental Table 2**
3) **Supplemental Table 3**

Supplemental Table 1: Baseline demographics and clinical characteristics for both centers.

Characteristics	KUMC (N=115)	UIHC (N=19)
**Average Age at starting tolvaptan (years)**	42.5 ± 11.9	36.2 ± 9.4
**Female**	57 (49.5%)	13 (68%)
**Average Height (centimeters)**	175.5 ± 10.7	173.3 ± 10.3
**Average Body mass index (kilograms/meters^2)**	29.2 ± 6.5	32.3 ± 6.7
**Black or African American/Non-Hispanic, Latine or Spanish Origin**	6 (5%)	
**Other/Hispanic, Latine or Spanish Origin**	5 (4%)	
**Other/ Non-Hispanic, Latine or Spanish Origin or Declined**	2 (2%)	1 (5%)
**White or Caucasian/Non-Hispanic, Latine or Spanish Origin**	102 (89%)	18 (95%)
**Family history of ADPKD**		
**Yes**	90 (78%)	18 (95%)
**No**	16 (14%)	1 (5%)
**Unsure**	9 (8%)	
**Comorbidities**		
**Hypertension**	99 (86%)	17 (89%)
**Liver Cysts**	46 (40%)	12 (63%)
**Brain Aneurysms**	2 (2%)	3 (16%)
**Diabetes Mellitus**	3 (3%)	1 (5%)
**Heart Disease**	10 (9%)	5 (26%)
**Use of RAASI**	88 (77%)	19 (100%)
**CKD Stage**		
**1**	18 (16%)	
**2**	33 (29%)	10 (53%)
**3a**	32 (28%)	5 (26%)
**3b**	19 (17%)	2 (11%)
**4**	13 (11%)	2 (11%)
**Average htTKV (mL/m) (N=115)**	963.8 ± 632.6	1047.8 ± 761.1
**Mayo ADPKD Imaging Classification (N=115)**		
**1A**	1 (1%)	
**1B**	15 (13%)	
**1C**	52 (45%)	11 (58%)
**1D**	27 (24%)	3 (16%)
**1E**	20 (17%)	4 (21%)
**Complications at baseline**		
**Kidney pain episodes**	16 (14%)	9 (47%)
**Cyst rupture/infection**	6 (5%)	3 (16%)
**UTI**	6 (5%	4 (21%

Supplemental Table 1: Baseline demographics and clinical characteristics of the University of Kansas Medical Center (KUMC) (N=115) and University of Iowa Hospital and Clinics (UIHC) (N=19).

Supplemental Table 2: Tolvaptan dosing trends for both centers.

Characteristics	KUMC (N=115)	UIHC (N=19)
**Dosing trend**		
Maintained on same dose	76 (66%)	15 (79%)
Required multiple changes to dose	39 (34%)	4 (21%)
**Starting tolvaptan dose**		
90 mg (AM)/ 30 mg (PM)	5 (4%)	1 (5%)
60 mg (AM)/ 30 mg (PM)	6 (5%)	
45 mg (AM)/ 15 mg (PM)	102 (89%)	17 (89%)
15 mg (AM)/ 15 mg (PM)		1 (5%
22.5 mg (AM)/ 7.5 mg (PM	2 (2%)	
**Ongoing tolvaptan dose**		
90 mg (AM)/ 30 mg (PM)	2 (2%)	
60 mg (AM)/ 30 mg (PM)	6 (5%)	
45 mg (AM)/ 15 mg (PM)	56 (49%)	9 (47%)
45 mg (AM)/ 7.5 mg (PM)	2 (2%)	
45 mg (AM)/ 0 mg (PM)	1 (1%)	
22.5 mg (AM)/ 7.5 mg (PM)	7 (6%)	
15 mg (AM)/ 15 mg (PM)		2 (11%)
22.5 mg (AM)/ 0 mg (PM	1 (1%)	
**Stopping tolvaptan dose**		
90 mg (AM)/ 30 mg (PM)	3 (3%)	1 (5%)
60 mg (AM)/ 30 mg (PM)	8 (7%)	
45 mg (AM)/ 15 mg (PM)	23 (20%)	5 (26%)
22.5 mg (AM)/ 7.5 mg (PM)	5 (4%)	
15 mg (AM)/ 15 mg (PM)		2 (11%)
22.5 mg (AM)/ 0 mg (PM)	1 (1%)	
**Reasons for stopping tolvaptan**		
Withdrew treatment due to adverse effects		
Polyuria and Polydipsia	10 (9%)	4 (21%)
Increase in LFTs	5 (4%)	1 (5%)
Brain fog and fatigue	2 (2%)	
Withdrew treatment due to unrelated factors		
Insurance coverage issues	5 (4%)	1 (5%)
Loss to follow-up	4 (3%)	1 (5%)
Unable to attend monthly labs	2 (2%)	
Poor medication compliance due to mental health issues	1 (1%)	
Withdrawn from treatment by physician		
Underwent renal transplantation	7 (6%)	1 (5%)
Started hemodialysis	1 (1%)	
Chronic disease flareup	2 (2%)	
Post-pregnancy/breastfeeding	1 (1%)	

Supplemental Table 2: Tolvaptan dosing trends and treatment withdrawal reasons of the KUMC (N=115) and UIHC (N=19) cohorts.

Supplemental Table 3:
Stopping dose and reason for withdrawing from tolvaptan treatment

Stopping Dosage and Reason for Stopping Tolvaptan	Statistic (N=134)
**Withdrew treatment due to adverse effects**	
**Polyuria and Polydipsia**	
22.5/0 mg	1 (0.75%)
45/15 mg	12 (9%)
60/30 mg	1 (0.75%)
**Increase in LFTs**	
15/15 mg	1 (0.75%)
22.5/7.5 mg	2 (1.5%)
45/15 mg	2 (1.5%)
60/30 mg	1 (0.75%)
**Brain fog and fatigue**	
45/15 mg	1 (0.75%)
60/30 mg	1 (0.75%)
**Withdrew treatment due to unrelated factors**	
**Insurance coverage issues**	
45/15 mg	6 (4.5%)
**Loss to follow-up**	
22.5/7.5 mg	1 (0.75%)
45/15 mg	3 (2.2%)
60/30 mg	1 (0.75%)
**Unable to attend monthly labs**	
45/15 mg	2 (1.5%)
**Poor medication compliance due to mental health issues**	
45/15 mg	1 (0.75%)

Supplemental Table 3: Dosage and reason for withdrawing from tolvaptan treatment for combined cohort (N=134).
